# Lengthening of the CHURC1 3' untranslated region by alternative polyadenylation is associated with the progression of acute myeloid leukemia

**DOI:** 10.1016/j.gendis.2024.101248

**Published:** 2024-02-28

**Authors:** Xi Hu, Panxiang Cao, Fang Wang, Huqin Zhang, Tong Wang, Hongxing Liu, Xiaoming Wu

**Affiliations:** a The Key Laboratory of Biomedical Information Engineering of Ministry of Education, School of Life Science and Technology, Xi'an Jiaotong University, Xi'an, Shaanxi 710049, China; b Division of Pathology and Laboratory Medicine, Hebei Yanda Lu Daopei Hospital, Langfang, Hebei 065201, China

Alternative polyadenylation (APA) is a post-transcriptional process that typically determines the length of mature mRNAs' 3' untranslated region (3'UTR). The global shortened 3'UTR alters mRNA stability, localization, and translation and contributes to cancer progression.^1^ The APA characteristics and its effects on acute myeloid leukemia (AML) remain to be comprehensively described. Here, we identified churchill domain containing 1 (CHURC1) as an up-regulated gene with a lengthened 3'UTR in CD34^+^ enriched AML cells compared with healthy hematopoietic stem and progenitor cells. As for APA regulation of CHURC1, we found that rs6745 and RNA binding protein TIA1 potentially contribute to the distal poly(A) site usage in *cis-* and *trans-*ways, respectively. Mechanistically, a lengthened CHURC1 3'UTR affected the apoptosis of AML cells by disrupting the expression of DICER1 by sponging miR-186–5p. The chemotherapeutic sensitivity of 8 drugs was associated with CHURC1 poly(A) site usage. Overall, our analysis indicated a previously undetected gene, CHURC1, that affected AML progression through 3'UTR length change resulting from APA. Data collection and detailed analysis methods were described in the supplementary material.

Due to the heterogeneity of AML blasts in different AML subtypes, we identified genes with shortened and lengthened 3'UTRs in t(8; 21) and non-t(8; 21) AML samples, respectively, compared with healthy CD34^+^ hematopoietic stem and progenitor cells following the approach used by Davis et al.^2^ We obtained 330 shortened and 129 lengthened genes in both t(8; 21) and non-t(8; 21) AML samples (Fig. S1; Fig. 1A). Combined with the results of differential expression analysis, we identified 114 shortened/lengthened genes with dysregulated expression in both t(8; 21) and non-t(8; 21) AML samples, including 36 up-regulated and 9 down-regulated genes with a lengthened 3'UTR and 31 up-regulated and 38 down-regulated genes with a shortened 3'UTR (cutoff values to select shortened/lengthened and up-/down-regulated genes were shown in the Supplementary Materials & Methods).

**Figure 1:**
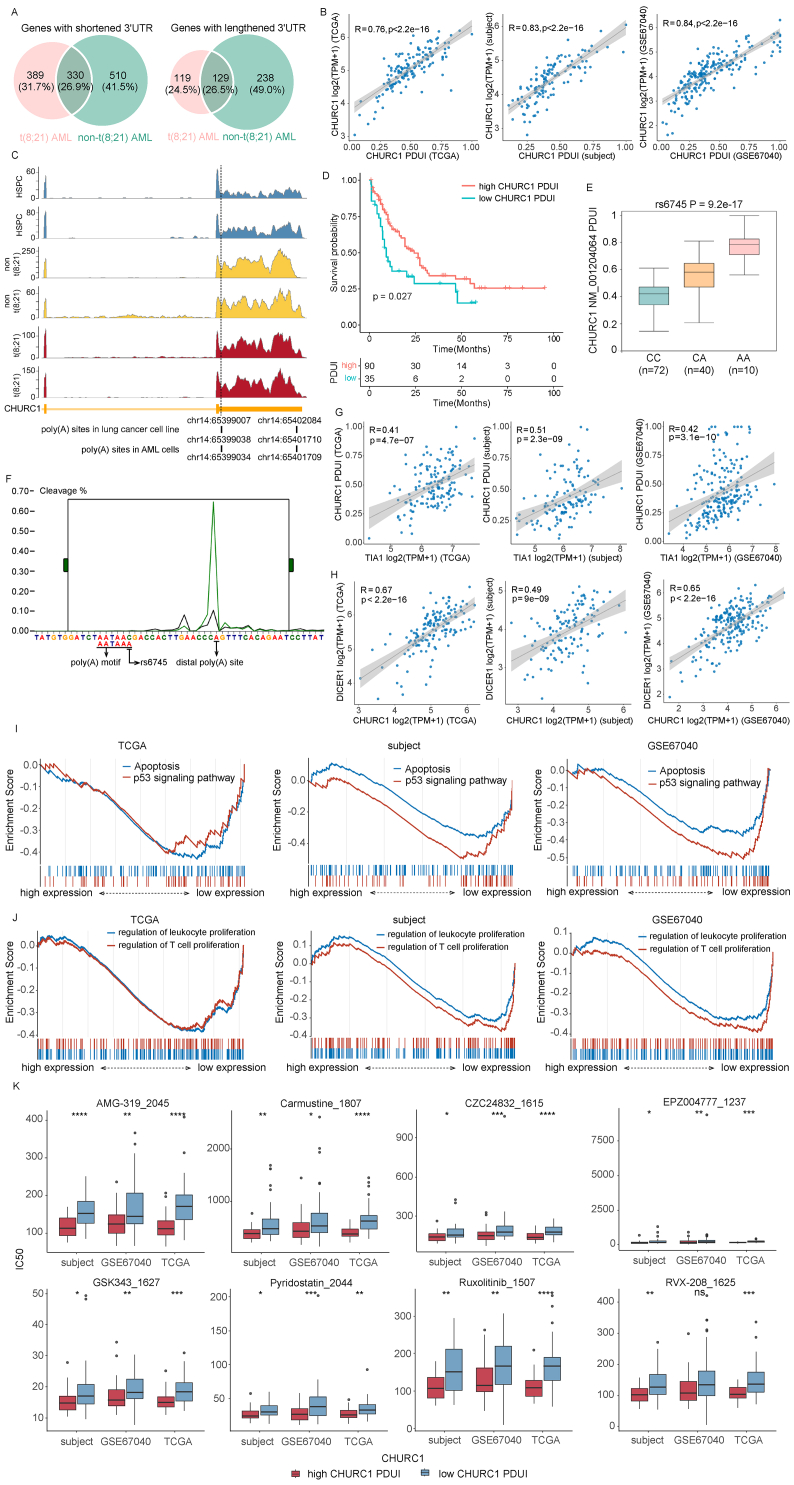
The 3'UTR change of CHURC1 caused by APA relates to AML. **(A)** The Venn diagrams showing the overlap of genes with shortened/lengthened 3'UTR in t(8; 21) and non-t(8; 21) AML compared with healthy CD34^+^ HSPCs. **(B)** The scatter plots showing the positive correlation between PDUI and log2(TPM+1) expression of CHURC1 in TCGA AML, GSE67040, and subject datasets. **(C)** RNA sequencing coverage tracks for 3'UTRs of CHURC1. The bottom tracks show the RefSeq gene structure, reported proximal and distal poly(A) sites in a lung cancer cell line, and reported proximal and distal poly(A) sites in AML. The dashed line indicates the predicted coordinate of the proximal poly(A) site in TCGA AML, GSE67040, and subject datasets. Healthy CD34^+^ HSPC samples are in blue; non-t(8; 21) AML samples are in yellow; and t(8; 21) AML samples are in red. **(D)** Kaplan-Meier estimates of overall survival time by CHURC1 PDUI in TCGA AML patients. **(E)** Distribution of CHURC1 PDUI for each genotype of rs6745 in TCGA AML samples. The allele A is associated with higher PDUI of CHURC1. **(F)** The predicted abundance changes of CHURC1 distal poly(A) site with rs6745 variants. The green lines represent APA distribution with allele A in rs6745. The black lines represent APA distribution with allele C in rs6745. The changed poly(A) motif by rs6745 is marked. **(G)** The scatter plots showing the positive correlation between log2(TPM+1) expression of TIA1 and PDUI of CHURC1 in TCGA AML, GSE67040, and subject datasets. **(H)** The scatter plots showing the positive correlation between log2(TPM+1) expression of CHURC1 and DICER1 in TCGA AML, GSE67040, and subject datasets. **(I)** Comparison of the enrichment of KEGG terms by GSEA of different CHURC1 PDUI. **(J)** Comparison of the enrichment of GO terms by GSEA of different CHURC1 PDUI. **(K)** The box plots showing IC_50_ of CHURC1 PDUI-related drugs in high and low PDUI groups of CHURC1. The CHURC1^high^ group is in red and the CHURC1^low^ group is in blue. ∗∗∗∗*P* < 0.0001, ∗∗∗*P* < 0.001, ∗∗*P* < 0.01, ∗*P* < 0.05. ns, not significant; 3'UTR, 3' untranslated region; APA, alternative polyadenylation; AML, acute myeloid leukemia; IC_50_, half maximal inhibitory concentration; GSEA, gene set enrichment analysis; HSPC, hematopoietic stem and progenitor cell; PDUI, percentage of distal poly(A) site usage index; R, Spearman's correlation coefficient; TPM, transcripts per million.

To identify APA-related genes in AML, we enrolled three independent RNA sequencing datasets of AML patients (labeled as subject, TCGA, and GSE67040 based on the data source). CHURC1, an up-regulated gene with a lengthened 3'UTR, showed the most significant correlation with RNA-level expression in all three datasets (Fig. S2; Fig. 1B). A previous study had verified that CHURC1 presented two poly(A) sites in a lung cancer cell line.^3^ The two sites also occurred in AML samples according to the 3'RNA sequencing data from GSE149237. Moreover, the same proximal poly(A) site was predicted in all three AML datasets (Fig. 1C). CHURC1 is expressed in various cells and higher in AML than most other cancers (Figs. S3–5), which indicates a potential effect of CHURC1 on AML. In the TCGA AML dataset, survival analysis revealed that low PDUI of CHURC1 was a risk factor for patient prognosis (Fig. 1D). Thus, we proposed that APA usage of CHURC1 might provide a new biomarker for AML. It needs further validation in more samples.

A previous study had performed apaQTL analyses across 32 cancers in TCGA.^4^ Among the *cis*-apaQTLs identified in TCGA AML data, we focused on rs6745, a single nucleotide polymorphism 15bp upstream of the CHURC1 distal poly(A) site, which produced a new canonical poly(A) signal AATAAA by a C > A change and was predicted to result in a 23.06-fold increase of the distal poly(A) site usage (Fig. 1E, F).

The 3'-end processing also depends on proteins binding to cis-regulatory motifs around poly(A) sites. We collected reported APA-related RNA binding proteins and GO terms associated with mRNA polyadenylation to evaluate the correlation between RNA binding proteins and poly(A) sites on CHURC1 in AML. We found that an RNA binding protein-coding gene TIA1 was down-regulated in t(8; 21) and non-t(8; 21) AML samples compared with healthy CD34^+^ hematopoietic stem and progenitor cells and its protein-level expression was relatively low in AML compared with other cancers (Fig. S6, 7). TIA1 interacted with U-rich motifs, which were enriched on the alternative 3'UTR of CHURC1 (Fig. S8). In the three AML datasets, the RNA-level expression of TIA1 correlated positively with the PDUI of CHURC1, indicating a distal poly(A) site preference by TIA1 (Fig. 1G). We also found a global 3'UTR lengthening in patients with high TIA1 expression (Fig. S9), which might provide a new therapeutic target to affect cell biological processes through transcriptional changes.

APA causes alternative 3'UTR which contains miRNA-binding motifs and may disrupt ceRNA cross-talk by sponging miRNAs. We found that AML-related miRNA miR-186–5p could combine on DICER1 and the alternative 3'UTR of CHURC1 (Fig. S10). DICER1 was up-regulated in AML patients and the CHURC1^high^ group (Fig. S11, 12). In the three AML datasets, the expression of CHURC1 and DICER1 were positively correlated (Fig. 1H). As a result, we proposed that DICER1 was a ceRNA partner of CHURC1 and the lengthening of CHURC1 3'UTR sponged miR-186–5p to increase the expression of DICER1. DICER1 is a highly conserved RNase III endonuclease essential for miRNA biogenesis and processing. Successful myeloid differentiation depends on the expression of a series of miRNAs. The dysregulation of the miR-186–5p/DICER1 axis might be one of the downstream mechanisms for the lengthened CHURC1 to affect AML progression.

To investigate the biological dysregulation related to poly(A) site usage of CHURC1 in AML, we divided each AML dataset into CHURC1^high^ and CHURC1^low^ groups according to the quantile PDUI value of CHURC1 and identified signaling pathways differentially activated between the two groups. According to the results of gene set enrichment analysis, cancer-related pathways such as apoptosis, p53 signaling, oxidative phosphorylation, antigen processing and presentation, and biological processes associated with T cell and leukocyte proliferation were significantly repressed in CHURC1^high^ groups (Fig. S13; Fig. 1I, J). We suggested that differential PDUI of CHURC1 may affect diverse biological processes related to AML progression.

To investigate potential chemotherapeutic drugs that may respond differently to poly(A) site usage in CHURC1, we predicted the half maximal inhibitory concentration (IC_50_) of 198 GDSC v2 drugs based on gene expression in the three AML datasets. Spearman's correlation was then assessed between the CHURC1 PDUI and IC_50_ values. We found that IC_50_ values of 8 drugs decreased with increasing CHURC1 PDUI (Fig. S14). A comparison of IC_50_ values between the CHURC1^high^ and CHURC1^low^ groups suggested that patients with higher PDUI were more likely to benefit from these drugs (Fig. 1K). The worse response to chemotherapeutics reminded a greater focus on the therapy and prognosis of patients with low CHURC1 PDUI.

This study concluded that CHURC1 APA site usage was related to AML progression and prognosis. Based on a published study on APA across 17 cancer types, PDUI of CHURC1 was positively related to its expression in 16 cancers.^5^ However, its function in cancers has not been demonstrated. This study had some limitations. The sample set of CD34^+^ enriched AML blasts and healthy hematopoietic stem and progenitor cells used to identify shortened/lengthened genes was small. Functional experiments are necessary to validate the mechanisms in AML proposed by data analyses. We only discussed the common APA occurring in t(8; 21) and non-t(8; 21) AML. Considering the differential molecular features of AML subtypes, specific APA characterization related to subtypes needs to be investigated.

## Ethics declaration

This study was approved by the ethics committee of Xi'an Jiaotong University (No. 2022–1081). Written informed consent for medical record review was obtained from all patients or their guardians following the Declaration of Helsinki.

## Author contributions

Xi Hu and Xiaoming Wu conceived the project and designed the experiments. Xi Hu planned all statistical analyses and drafted the manuscript. Panxiang Cao processed RNA sequencing data. Fang Wang and Tong Wang collected clinical samples and diagnosed cases. Huqin Zhang and Hongxing Liu edited the manuscript.

## Conflict of interests

The authors declare no competing financial interests.

## Funding

This work was supported by the Technology Innovation Leading Program of Shaanxi, China (No. 2023KXJ-219) and the Science and Technology Program of Shaanxi Province, China (No. 2022LL-ZD-01HZ).
