# Supplementary material for: Lengthening of the CHURC1 3' untranslated region by alternative polyadenylation is associated with the progression of acute myeloid leukemia
Source: Genes Dis. 2024 Feb 28;12(1):101248. doi: 10.1016/j.gendis.2024.101248 (PMC12053556; doi:10.1016/j.gendis.2024.101248)
Supplement: Multimedia component 1 [file mmc1.docx]

**Supplementary Materials & Methods**

**Study subject**

A cohort of 123 patients with a confirmed diagnosis of AML in Hebei Yanda Lu Daopei Hospital was enrolled in this study. Total RNA was extracted from the bone marrow samples, and paired-end sequencing with a read length of 150bp was performed on the Illumina HiSeq 2500 platform. The dataset was labeled as “subject” in the paper.

**Public data collection**

Public RNA sequencing data were collected from the Gene Expression Omnibus (GEO) database (<https://www.ncbi.nlm.nih.gov/geo/>). RNA sequencing data from CD34+ enrichment cells of 8 AML samples (4 patients with t(8:21) translocation and 4 without) and 5 healthy HSPC controls were obtained from GSE149237. RNA sequencing data from 208 AML bone marrow samples were downloaded from GSE67040 (the dataset was labeled as “GSE67040” in the paper).

**Data preprocessing**

All RNA sequencing data were aligned with the human genome (GRCh37/hg19) using HISAT2 v2.1.0.^1^ The number of mapping reads of each sample was counted using SAMtools v1.9.^2^ Gene RNA expression was quantified using featureCounts v.2.0.1.^3^ Raw count values were converted into transcripts per million (TPM) values. The sorted BAM files were converted into WIG formats using BEDTools v2.25.0.^4^ DaPars v.2^5^ was used to identify the percentage of distal poly(A) site usage index (PDUI) of each AML sample based on the 3’UTR annotation available in the UCSC table browser (https://genome.ucsc.edu). PDUI ranges from 0 to 1; a greater PDUI represents more transcripts using the distal APA site. In addition, APA usage and RNA expression data of 144 AML patients were downloaded from the TC3A database^6^ and TCGA database (https://portal.gdc.cancer.gov/), respectively (the dataset was labeled as “TCGA” in the paper). Genes were retained if read counts > 10 in more than 30% of samples. APA events and samples with the missing rate of PDUI values < 30% were selected.

**Identification of APA-related genes in AML**

The difference in APA usage between AML and healthy HSPC was evaluated using $\Delta PDUI ({MeanPDUI}_{AML}-{MeanPDUI}_{HSPC})$. The Wilcoxon rank-sum test was used to analyze the PDUI distribution between unpaired groups. Genes with $P value<0.05$and $\Delta PDUI>0.1$ or $\Delta PDUI<-0.1$ were considered significantly lengthened or shortened in AML, respectively. Differentially expressed genes in AML compared with healthy HSPC were analyzed using the DESeq2 package. Genes with $FDR<0.05$ and $Fold change>1.2$ or $Fold change<0.8$ were considered significantly up-regulated or down-regulated in AML, respectively. The correlation between PDUI and corresponding gene expression in AML samples was assessed using Spearman’s correlation analysis with the threshold of $|rho|>0.3$and $P value<0.05$. Ultimately, genes with changed 3’UTR that were related to dysregulated expression were defined as APA-related genes in AML in this study.

**Selection of cis-apaQTLs in AML**

Cis-apaQTLs identified in TCGA AML samples were obtained from the SNP2APA database.^7^ We extracted apaQTLs within the 3’UTR of APA-related genes. Using sequences under different alleles of cis-apaQTL as input, we used the Variants module of APARENT to predict the abundance change of isoforms with APA sites.^8^

**Identification of RBPs that regulate APA in AML**

We collected 102 APA-related RBPs from a published paper^5^ and GO terms associated with mRNA polyadenylation (RNA polyadenylation, mRNA alternative polyadenylation, and regulation of mRNA polyadenylation) to evaluate the potential effects of RBPs on APA sites of CHURC1 in AML. The RBP binding regions on CHURC1 alternative 3’UTR were predicted using the RBPsuite web server (<http://www.csbio.sjtu.edu.cn/bioinf/RBPsuite/>). The RBPsuite divides the input sequence into segments of length 101 without overlap and displays the score of each segment with the predicted RBP.

AML samples in each dataset were divided into TIA1^high^ and TIA1^low^ groups according to the quantile TPM value of TIA1. In detail, samples with $TPM\geq Q3$ were taken as the TIA1^high^ group; samples with $TPM\leq Q1$ were taken as the TIA1^low^ group. Shortened/lengthened genes were identified using the method described above to analyze TIA1’s global effects on APA in AML.

**Establishment of ceRNA network**

We obtained AML-related miRNAs from GeneCards (https://www.genecards.org/), miR2Disease (http://www.mir2disease.org/), and HMDD v3.2 database (http://www.cuilab.cn/hmdd). The miRNA binding regions on mRNAs predicted by 7 algorithms were downloaded from the ENCORI database (collected by 23 May 2022).^9^ AML-related miRNAs that were predicted to bind to the alternative 3’UTR of CHURC1 by more than 2 algorithms were filtered. The target genes of these miRNAs, the expression of which also conformed to the negative regulation rule, were selected as candidate ceRNAs to construct the ceRNA network.

**Gene set enrichment analysis (GSEA)**

AML samples in each dataset were divided into CHURC1^high^ and CHURC1^low^ groups according to the quantile PDUI value of CHURC1. In detail, samples with $PDUI\geq Q3$ were taken as the CHURC1^high^ group; samples with $PDUI\leq Q1$ were taken as the CHURC1^low^ group. Differential expression analysis was used to calculate the log2(Fold change) of gene expression between the two groups, then GSEA was performed using the clusterProfiler package. Gene sets were filtered with a maximum gene set size of 1000 and minimum of 10. Gene sets with $P value<0.05$, $FDR<0.25$, and $\left| NES \right|>1$ in all three AML datasets were significantly enriched.

**Drug sensitivity analysis**

We used the calcPhenotype function in the oncoPredict package to predict the chemosensitivity in AML. It built a ridge regression model for each drug based on data from the Genomics of Drug Sensitivity in Cancer (GDSC) v2 (https://www.cancerrxgene.org/). Then, gene expression values were inputted into the models to predict half maximal inhibitory concentration (IC50) of each drug in the three AML datasets. Default values were used for all parameters of the function. The correlation between the CHURC1 PDUI and the estimated half maximal inhibitory concentration (IC50) of each chemotherapeutic drug was evaluated using Spearman’s correlation analysis with a significance threshold of $FDR<0.05$. The difference in IC50 of PDUI-related drugs between the CHURC1^high^ and CHURC1^low^ groups was assessed using the Wilcoxon rank-sum test.

**Statistical analysis**

All statistical analyses were performed using R version 4.2.3. Kaplan-Meier analysis and log-rank test were used to compare survival by different levels of PDUI or gene expression, which were divided by quartile. The survival analysis was performed using the Survival package. The Benjamini & Hochberg adjustment was used for the false discovery rate (FDR). A $P value<0.05$ was considered significant for all statistical analyses unless otherwise specified.

**Supplementary Figures**


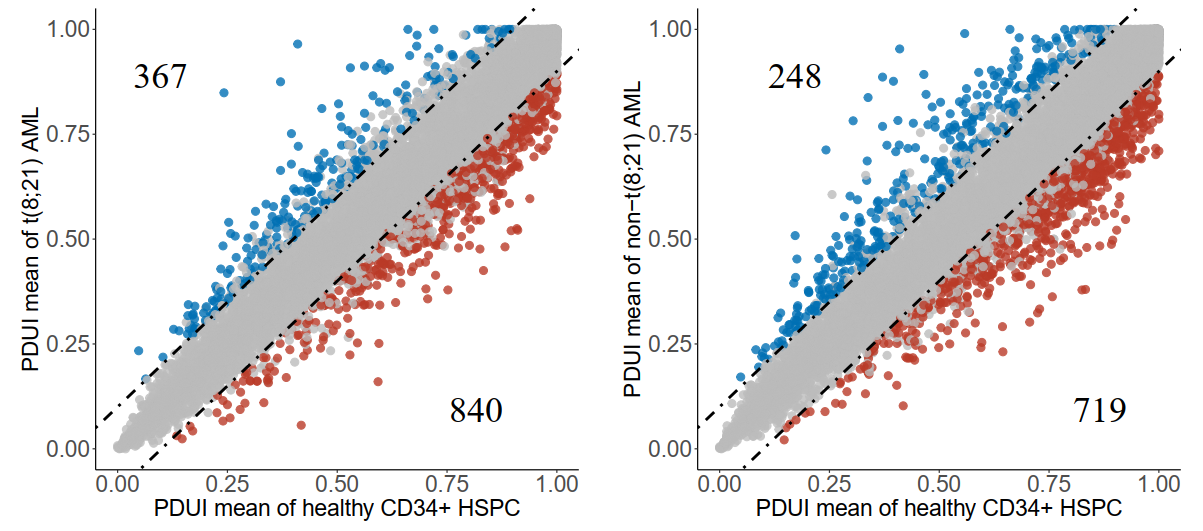


**Fig.S1** **Identification of genes with shortened/lengthened 3’UTR in AML.** Scatter plots showing the change of PDUI in t(8;21) AML and non-t(8;21) AML compared to healthy CD34+ HSPC samples. Blue dots indicate significantly lengthened genes (∆PDUI > 0.1 and P value < 0.05); red dots indicate significantly shortened genes (∆PDUI < -0.1 and P value < 0.05). Dashed lines represent |∆PDUI|=0.1.


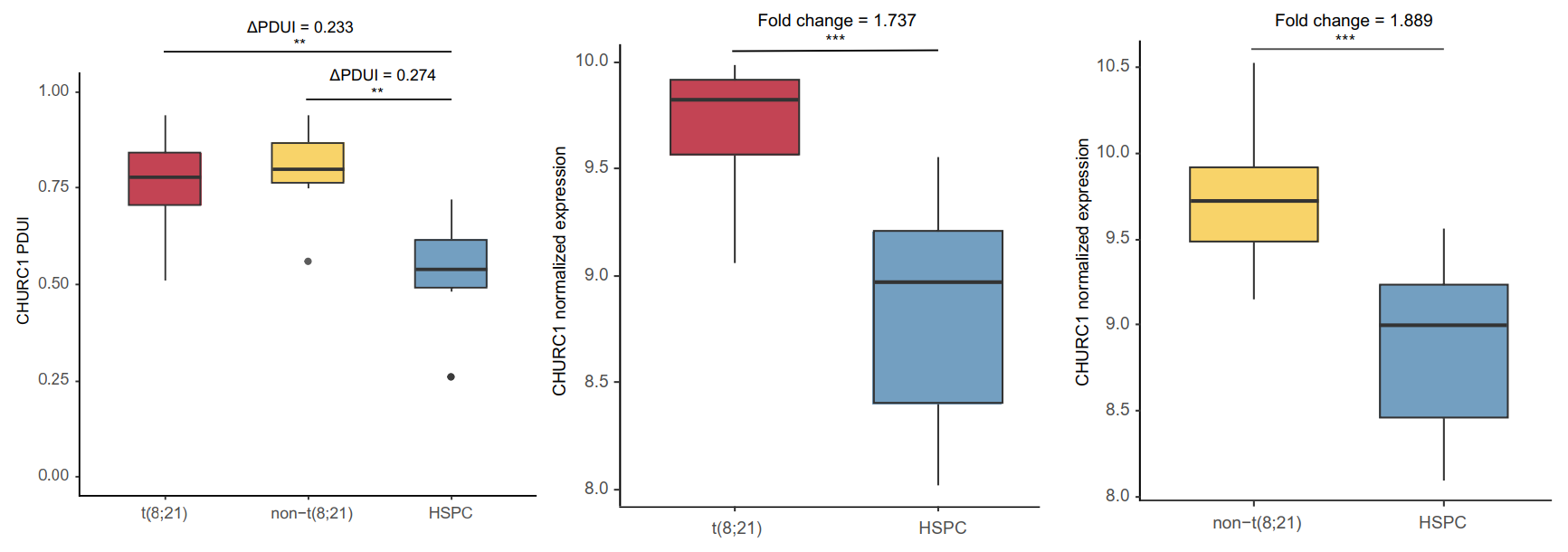


**Fig.S2 The comparation of PDUI and expression of CHURC1 between AML and CD34+ HSPCs.** Box plots show PDUI and log2(TPM+1) expression of CHURC1 in t(8;21) AML, non-t(8;21) AML and healthy CD34+ HSPC. Healthy CD34+ HSPC samples are in blue; non-t(8;21) AML samples are in yellow; t(8;21) AML samples are in red. *** P-value < 0.001, ** P-value < 0.01.


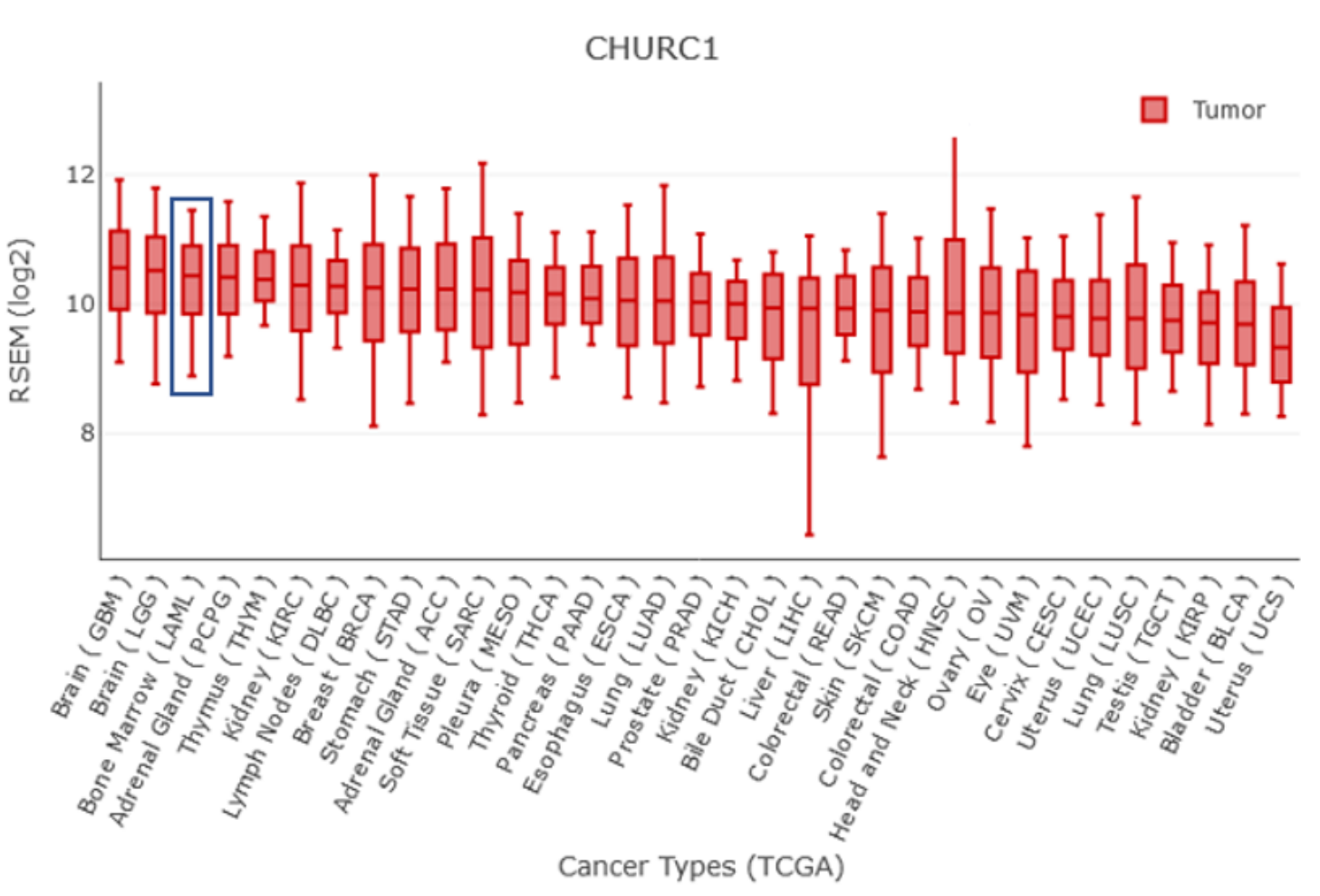


**Fig.S3 RNA level expression of CHURC1 across TCGA cancers from the GEDS web server.** ^10^


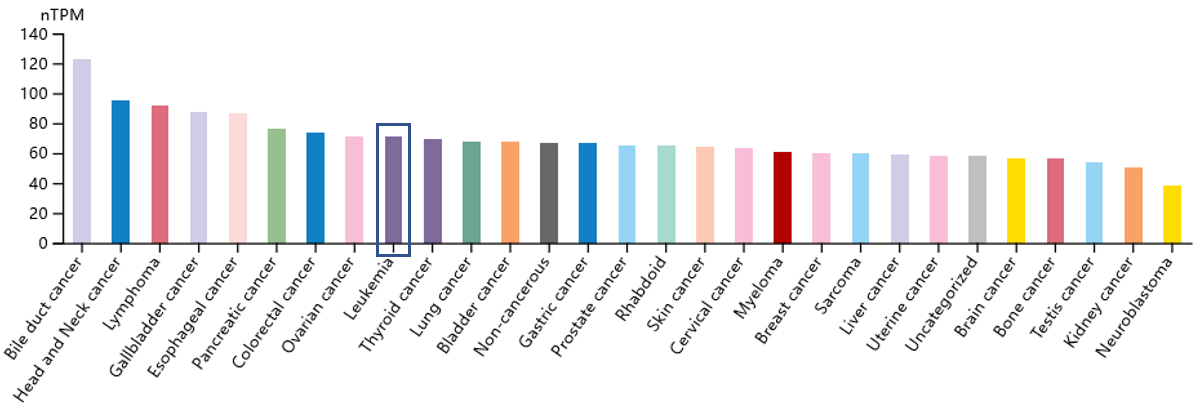


**Fig.S4 RNA level expression of CHURC1 across cancer cell lines from The Human Protein Atlas database (https://www.proteinatlas.org/).** AML cell lines are contained in Leukemia.


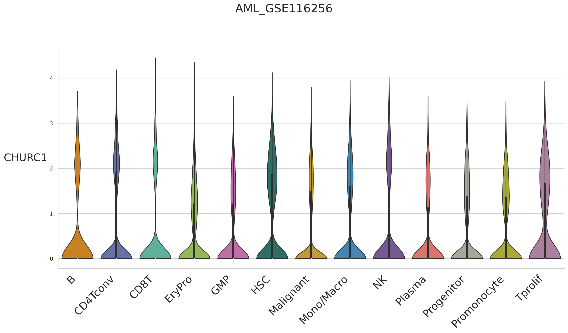


**Fig.S5 RNA level expression of CHURC1 across different types of cells from single-cell AML_GSE116256 dataset in the TISCH2 database (http://tisch.comp-genomics.org/).**


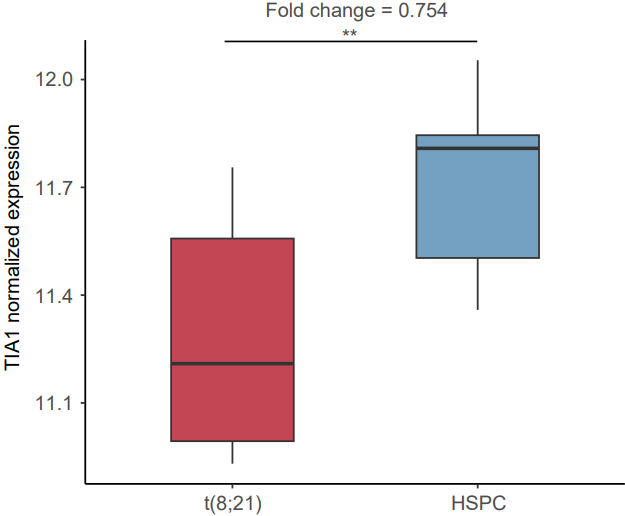

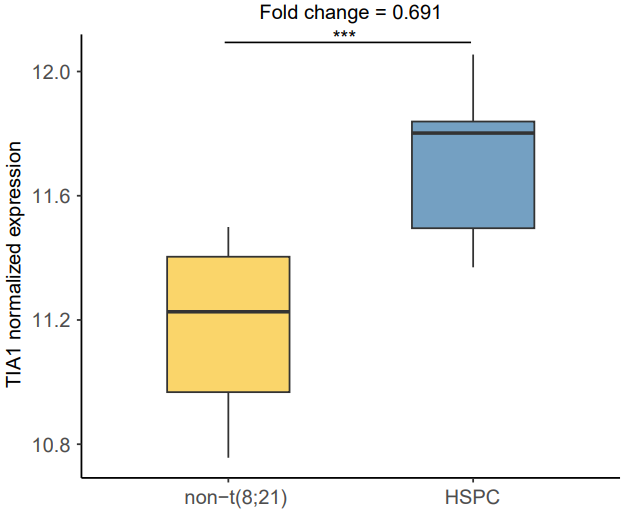


**Fig.S6 Box plots showing expression of TIA1 in t(8;21) AML (left) and non-t(8;21) AML (right) compared to healthy CD34+ HSPC.** Healthy CD34+ HSPC samples are in blue; non-t(8;21) AML samples are in yellow; t(8;21) AML samples are in red. *** P-value < 0.001, ** P-value < 0.01.


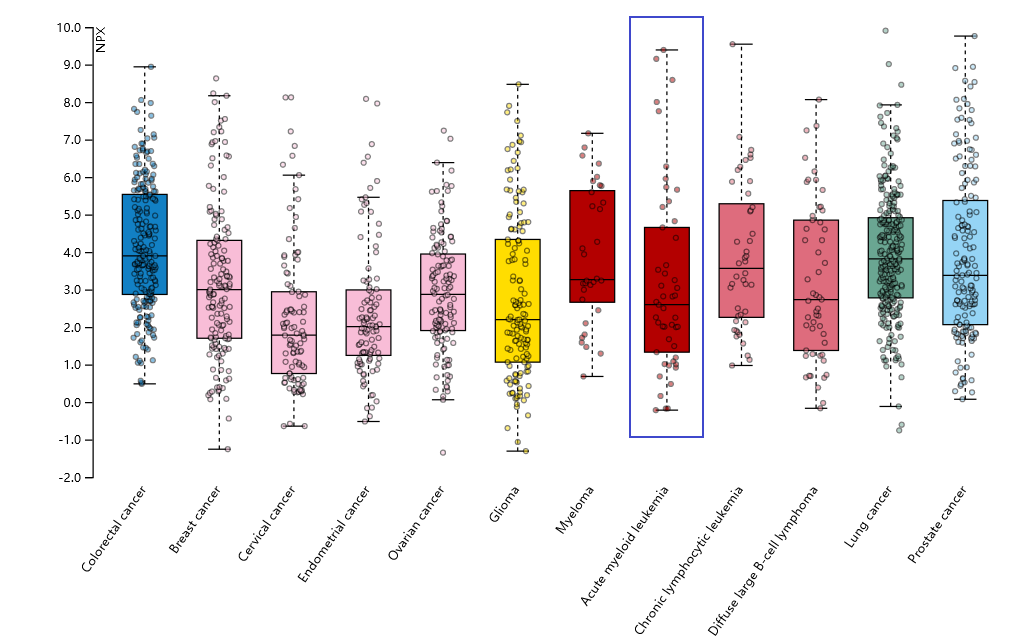


**Fig.S7 Protein concentrations of TIA1 in the pan-cancer cohort from The Human Protein Atlas database.**


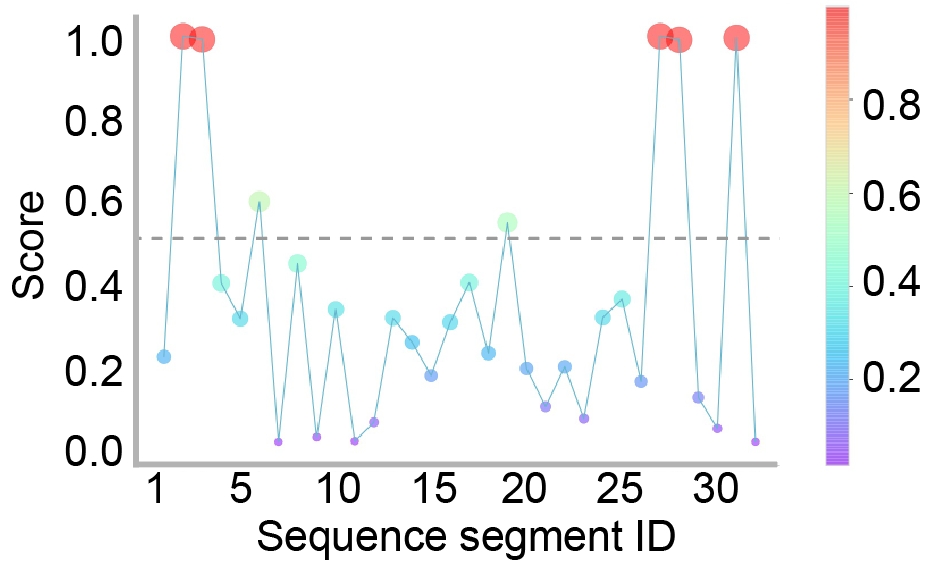


**Fig.S8 The location and scores of U-rich motifs predicted by RBPsuite web server on the 3’UTR of CHURC1.** Each dot indicates the TIA1-binding score on each 101bp segment. The dashed line represents Score=0.5.


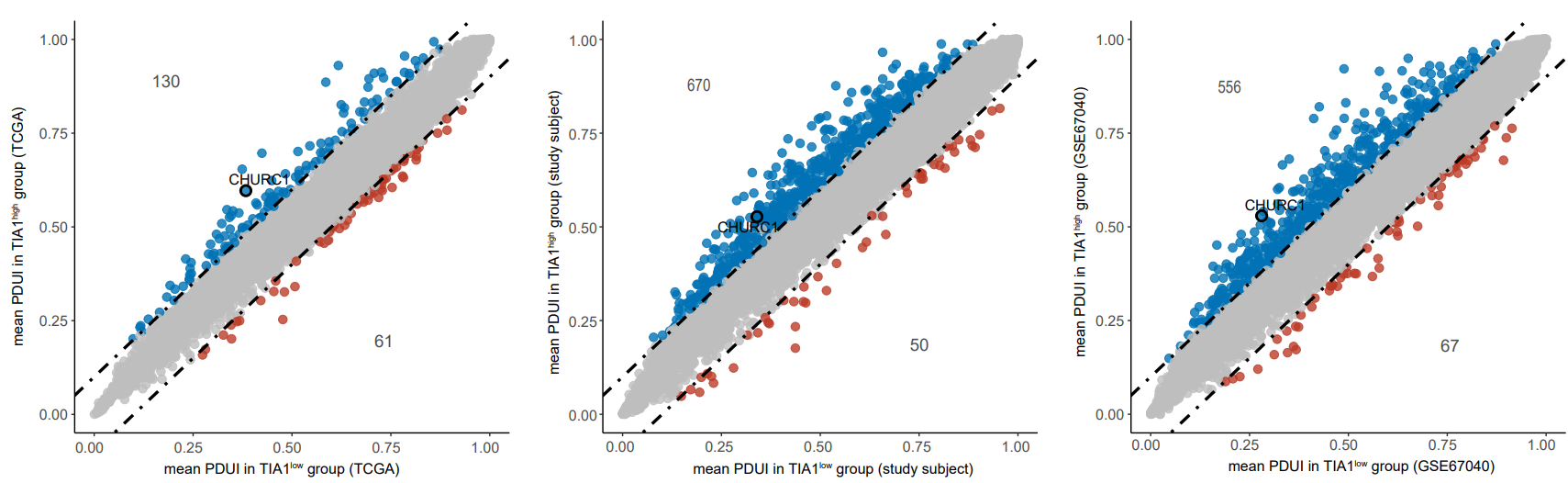


**Fig.S9 Scatter plots showing the change of PDUI between the TIA1^high^ and TIA1^low^ groups in TCGA AML, GSE67040, and subject datasets.** Blue dots indicate significantly lengthened genes ($\Delta PDUI>0.1$ and $P value<0.05$); red dots indicate significantly shortened genes ($\Delta PDUI<-0.1$ and $P value<0.05$). Dashed lines represent $|\Delta PDUI|=0.1$.


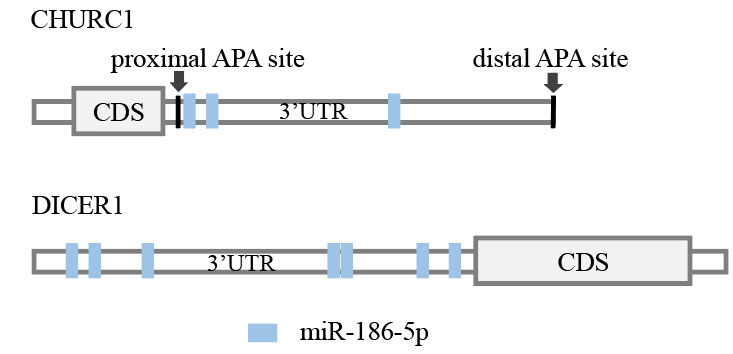


**Fig.S10 Schematic showing predicted binding sites of miR-186-5p (blue) within 3’UTRs of CHURC1 and DICER1.**


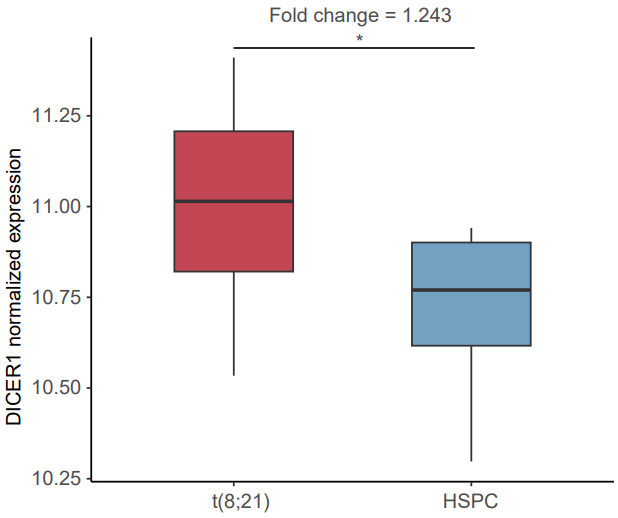

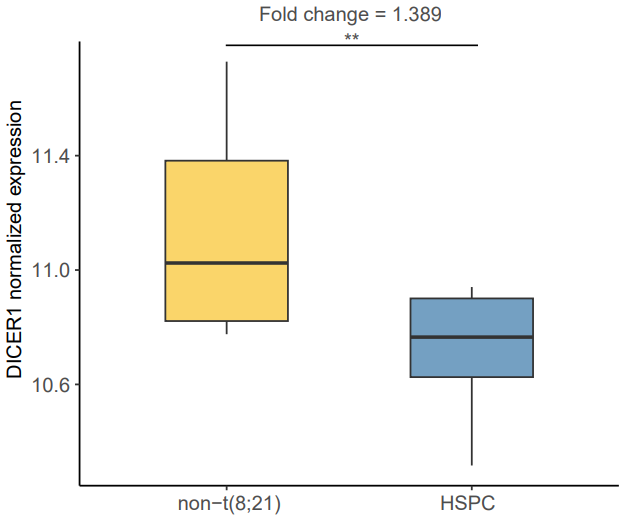


**Fig.S11 Box plots showing expression of DICER1 in t(8;21) AML (left) and non-t(8;21) AML (right) compared to healthy CD34+ HSPC.** Healthy CD34+ HSPC samples are in blue; non-t(8;21) AML samples are in yellow; t(8;21) AML samples are in red. ** P-value < 0.01, * P-value < 0.05.


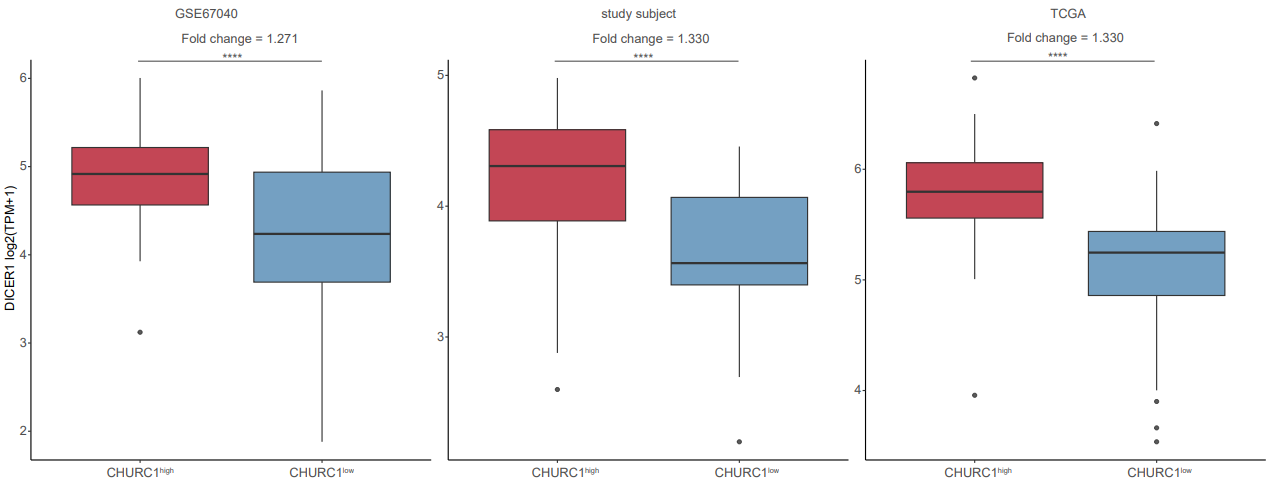


**Fig.S12 Box plots showing expression of DICER1 in CHURC1^high^ and CHURC1^low^ groups in TCGA AML, GSE67040, and subject datasets.** **** P-value < 0.0001.


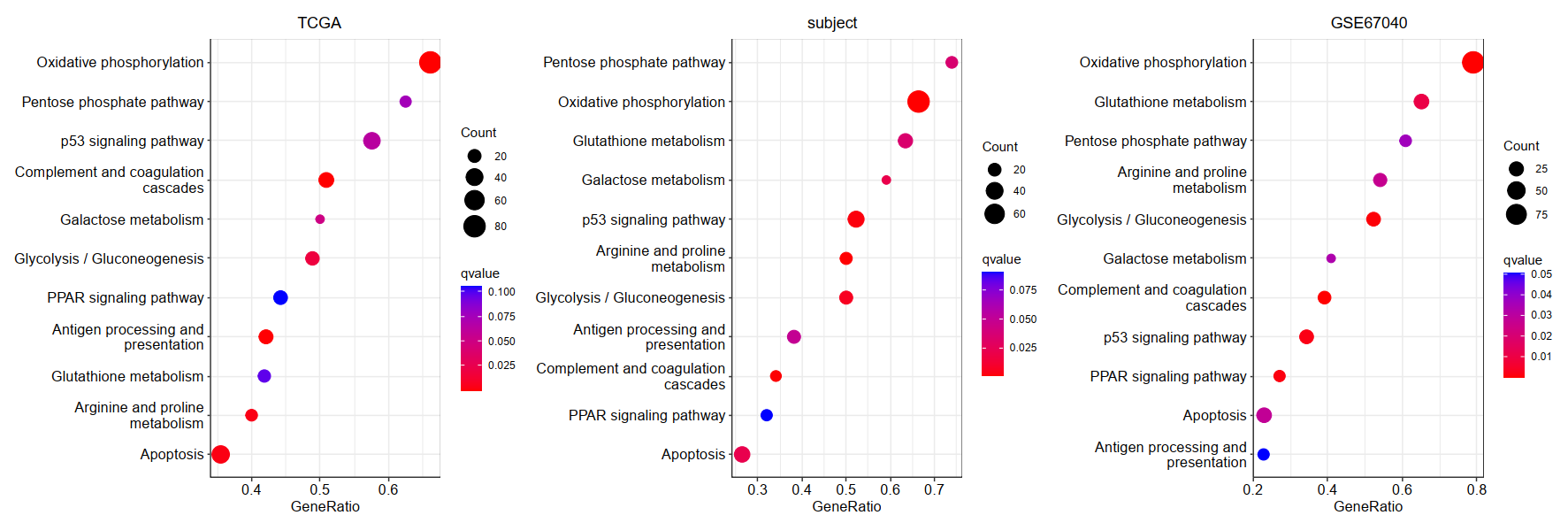


**Fig.S13 GSEA analysis of high and low PDUI of CHURC1 in TCGA AML, GSE67040, and subject datasets.** Common cancer-related KEGG terms enriched in different groups of CHURC1 PDUI.


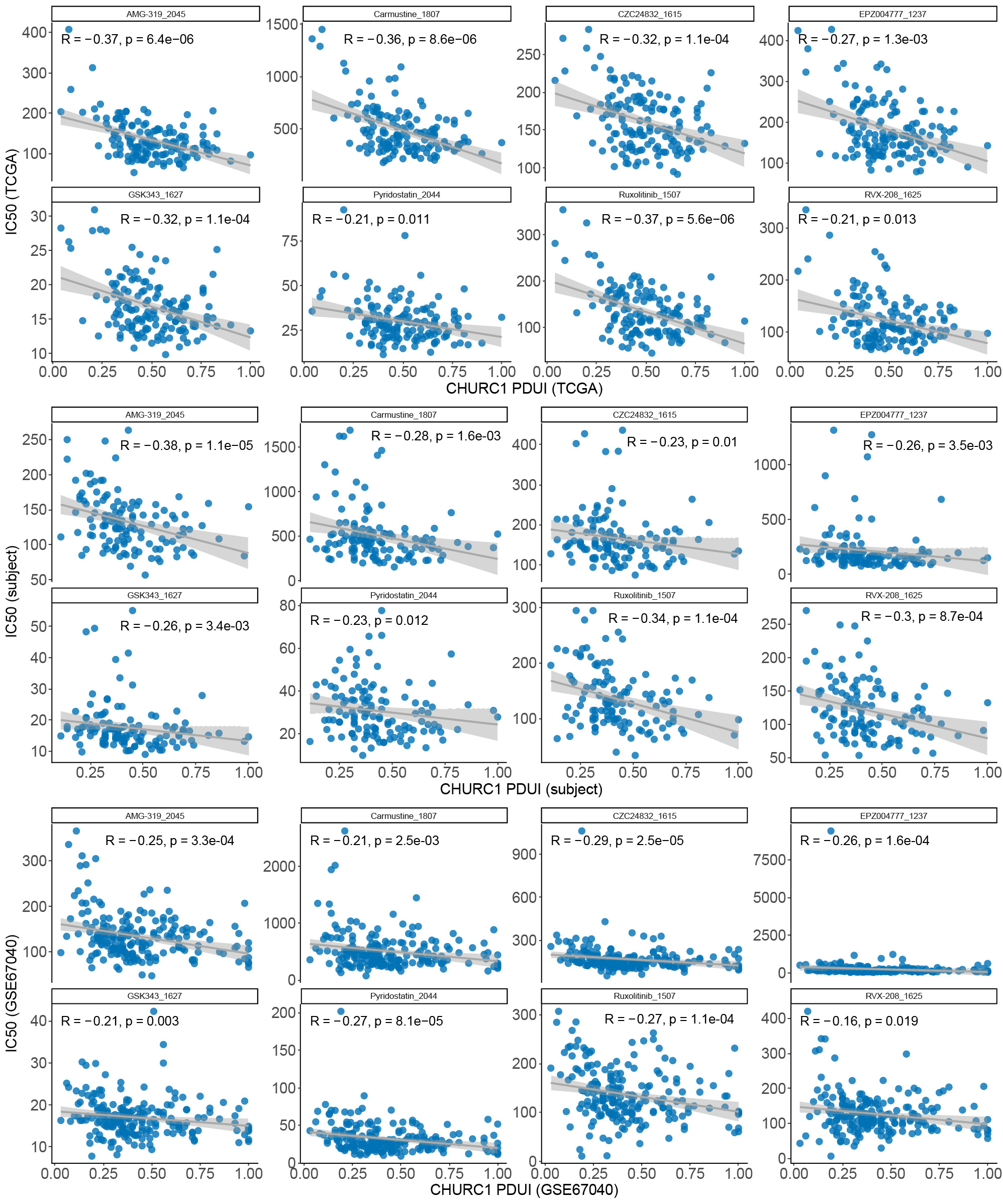


**Fig.S14 Drug sensitivity is related to the PDUI of CHURC1 in TCGA AML, GSE67040, and the subject datasets.** Scatter plots showing the negative correlation between CHURC1 PDUI and IC50 of drugs. R: Spearman’s correlation coefficient.

**Reference**

1. Kim D, Paggi JM, Park C, Bennett C, Salzberg SL. Graph-based genome alignment and genotyping with HISAT2 and HISAT-genotype. *Nat Biotechnol.* 2019;37(8):907-915.

2. Li H, Handsaker B, Wysoker A, et al. The Sequence Alignment/Map format and SAMtools. *Bioinformatics.* 2009;25(16):2078-2079.

3. Liao Y, Smyth GK, Shi W. featureCounts: an efficient general purpose program for assigning sequence reads to genomic features. *Bioinformatics.* 2014;30(7):923-930.

4. Quinlan AR, Hall IM. BEDTools: a flexible suite of utilities for comparing genomic features. *Bioinformatics.* 2010;26(6):841-842.

5. Li L, Huang KL, Gao Y, et al. An atlas of alternative polyadenylation quantitative trait loci contributing to complex trait and disease heritability. *Nat Genet.* 2021;53(7):994-1005.

6. Feng X, Li L, Wagner EJ, Li W. TC3A: The Cancer 3' UTR Atlas. *Nucleic Acids Res.* 2018;46(D1):D1027-D1030.

7. Yang Y, Zhang Q, Miao YR, et al. SNP2APA: a database for evaluating effects of genetic variants on alternative polyadenylation in human cancers. *Nucleic Acids Res.* 2020;48(D1):D226-D232.

8. Bogard N, Linder J, Rosenberg AB, Seelig G. A Deep Neural Network for Predicting and Engineering Alternative Polyadenylation. *Cell.* 2019;178(1):91-106 e123.

9. Li JH, Liu S, Zhou H, Qu LH, Yang JH. starBase v2.0: decoding miRNA-ceRNA, miRNA-ncRNA and protein-RNA interaction networks from large-scale CLIP-Seq data. *Nucleic Acids Res.* 2014;42(Database issue):D92-97.

10. Xia M, Liu CJ, Zhang Q, Guo AY. GEDS: A Gene Expression Display Server for mRNAs, miRNAs and Proteins. *Cells.* 2019;8(7).
